# Impact of infection prevention precautions on adenoviral infections during the coronavirus disease 2019 (COVID-19) pandemic: Experience of a tertiary-care hospital in Singapore

**DOI:** 10.1017/ice.2020.1365

**Published:** 2020-12-10

**Authors:** Liang En Wee, Edwin Philip Conceicao, Jean Xiang Ying Sim, May Kyawt Aung, Indumathi Venkatachalam

**Affiliations:** 1 SingHealth Infectious Diseases Residency, Singapore; 2 Department of Infectious Diseases, Singapore General Hospital, Singapore; 3 Department of Infection Prevention and Epidemiology, Singapore General Hospital, Singapore

*To the Editor—*We read with interest the article by Wong et al^1^ describing the impact of infection control measures introduced during the COVID-19 pandemic on nosocomial transmission of influenza and respiratory syncytial virus (RSV). Although the authors experienced zero nosocomial transmission of enveloped respiratory viral infections (RVIs), nonenveloped RVIs, such as adenoviruses, may pose greater challenges in infection prevention. Indeed, given the relative hardiness of the nonenveloped adenoviruses, evidence of contamination with adenoviruses has been reported in healthcare environments^2^ as well as on the outer surface of medical masks used by healthcare workers (HCWs).^3^ Given the potential of adenoviruses to persist on inanimate surfaces and retain infectivity and the limited effectiveness of alcohol-based hand disinfection in eliminating this viral pathogen,^4^ adenovirus outbreaks can occur even in the setting of good hand hygiene compliance.^5^ Although adenoviruses generally cause self-limiting disease in immunocompetent hosts, infections in vulnerable populations, such as transplant recipients and neonates, can be potentially devastating.^6^ Therefore, we sought to determine whether infection prevention measures introduced during the COVID-19 pandemic could potentially influence transmission of adenoviral infections.

In Singapore, a Southeast Asian city-state, various infection prevention measures were implemented across all public hospitals soon after the first reported case of COVID-19 at the end of January 2020. At our institution, the largest acute- and tertiary-care hospital in Singapore, an integrated strategy was introduced from February 2020 to mitigate healthcare-associated transmission of SARS-CoV-2.This strategy focused on universal masking for all HCWs, adherence to basic infection prevention measures including hand hygiene, and improved segregation of patients with respiratory symptoms.^7^ Before the pandemic, patients were predominantly nursed in multi-bedded open-plan wards. During the pandemic, patients with respiratory symptoms were segregated in dedicated wards with reduced bed density, and HCWs used disposable gloves, gowns, eye protection, and N95 respirators until COVID-19 was excluded.^7^ The combined infection prevention bundle successfully prevented patient–HCW transmission of SARS-CoV-2 and reduced healthcare-associated influenza transmission,^7,8^ but the impact on transmission of adenoviral infections was not specifically assessed. Our institution provides neonatal services, as well as an active bone-marrow and solid-organ transplant program. During the pandemic, our institution continued to function and accept patients as usual. Over a 9-month study period during the COVID-19 pandemic from February to October 2020, all symptomatic inpatients were tested for SARS-CoV-2; adenovirus was also tested as 1 of the 16 common circulating RVIs on our institution’s multiplex PCR panel. Adenoviral infections were categorized as healthcare associated if the RVI was identified beyond the maximum incubation period (14 days) from admission. Comparisons of adenoviral infection rates (both community-acquired/healthcare-associated) during the pandemic period were made with the corresponding period before the pandemic (January 2015–January 2020) using the incidence rate ratio (IRR) method. Our institution’s institutional review board approved this study with a waiver of informed consent.

Before the pandemic, the incidence of healthcare-associated adenoviral infections was 0.40 cases per 10,000 patient days (94 cases; 2,368,810 patient days). After the implementation of the integrated infection prevention bundle in February 2020, the incidence of healthcare-associated adenoviral infections fell to 0.03 cases per 10,000 patient days (1 case; 349,130 patient days), a statistically significant decrease (IRR, 0.07; 95% confidence interval [CI], 0.01–0.41; *P* < .05). The sole case of potential healthcare-associated infection during the pandemic occurred in a returning COVID-19–positive traveler, who was isolated in a negative-pressure airborne-infection isolation room (AIIR) in our institution’s isolation ward for 21 days (Supplementary Fig. 1a online). He was retested for RVIs due to new-onset respiratory symptoms. Although respiratory specimens at admission were negative for adenovirus, repeated specimens at day 21 were positive. All HCWs used disposable gloves, gowns, eye protection, and N95 respirators, changed at each patient encounter. Hand hygiene compliance remained at 100% and compliance with personal protective equipment use was maintained at ≥90%.^9^ The patient was confined to the same AIIR throughout, and our isolation ward had a strict no-visitor policy, although one-way transfer of personal items into the AIIR was allowed to mitigate the psychological effects of prolonged isolation. Though a fomite source is possible, it could not be conclusively proven. The mortality rate among all patients with adenoviral infections was 5.53% (42 deaths among 759 cases) (Supplementary Fig. 1b online). From March to October 2020, there were zero cases of healthcare-associated adenoviral infections over a sustained 8-month period, an observation unprecedented in the preceding 5 years of surveillance (Supplementary Fig. 2 online).

This significant decrease in healthcare-associated transmission of adenoviral infections occurred despite increased driving pressure from admissions with community-acquired adenoviral infections in the initial pandemic phase (Fig. 1a). During the first 4 months of the pandemic (February–May 2020), the incidence of community-acquired adenoviral infections was higher than baseline, at 2.23 cases per 1,000 admissions (49 cases, 21,881 admissions), compared with a prepandemic rate of 1.19 cases per 1,000 admissions (162 cases; 135,124 admissions) in the corresponding period (February–May 2015–2019), a statistically significant increase (IRR, 1.87; 95% CI, 1.33–2.59; *P* < .001). Only after the imposition of a national “lockdown” period from April 7 through June 1, 2020, was there a significant decrease in the incidence of community-acquired adenoviral infections among admissions: the pandemic period (0.06 cases per 1,000 admissions, 13 cases, and 210,237 admissions) versus the prepandemic period from June through October 2015–2019 (1.32 cases per 1,000 admissions, 226 cases, and 171,408 admissions) (IRR, 0.05; 95% CI, 0.02–0.08; *P* < .001). This finding contrasts with observations made for enveloped RVIs, in which the introduction of community-wide public health measures, such as social distancing and the universal use of face coverings, was associated with a significant decrease in influenza-incidence in the general population.^10^

**Fig. 1.**
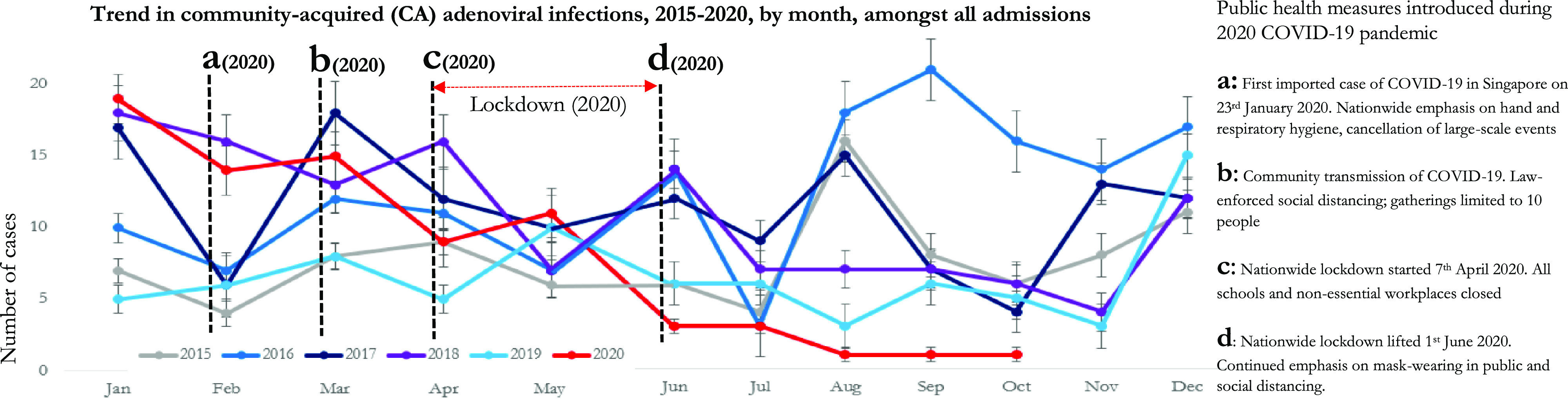
Trends in community-acquired (CA) adenoviral infections amongst all admissions to a tertiary hospital in Singapore.

The key finding of this study is that infection prevention precautions have been effective in reducing healthcare-associated transmission of adenoviral infections during the COVID-19 pandemic, despite the increased rate of community-acquired adenoviral infections in the corresponding period. However, the sole case of healthcare-associated transmission of adenoviral infection in a COVID-19–positive individual, despite full isolation precautions, demonstrates the significant infection prevention challenges posed by this hardy nonenveloped viral pathogen.

## Supplementary material

For supplementary material accompanying this paper visit https://doi.org/10.1017/ice.2020.1365. click here to view supplementary material
