# Supplementary material for: Impact of infection prevention precautions on adenoviral infections during the coronavirus disease 2019 (COVID-19) pandemic: Experience of a tertiary-care hospital in Singapore
Source: Infect Control Hosp Epidemiol. 2020 Dec 10:1–2. doi: 10.1017/ice.2020.1365 (PMC8770837; doi:10.1017/ice.2020.1365)
Supplement: Supplementary file 1 [file icesup.zip › S0899823X20013653sup001.docx]

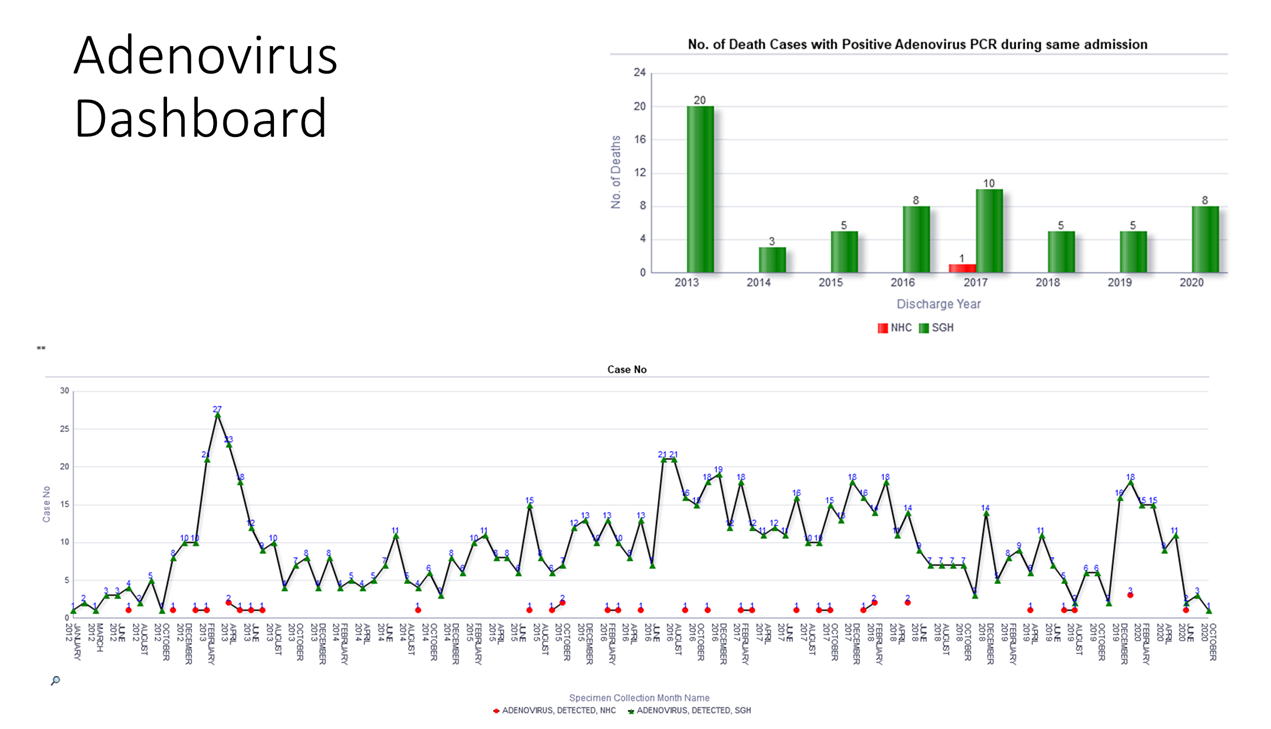

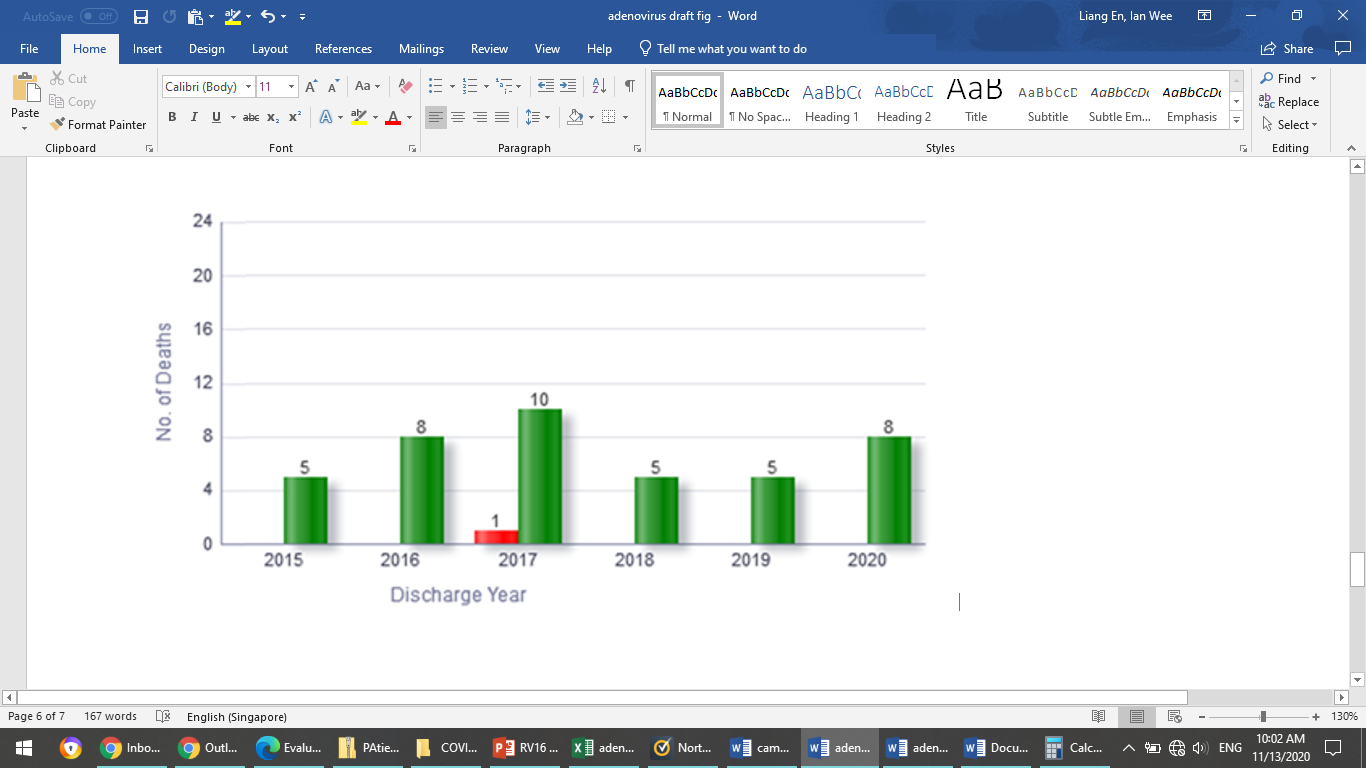

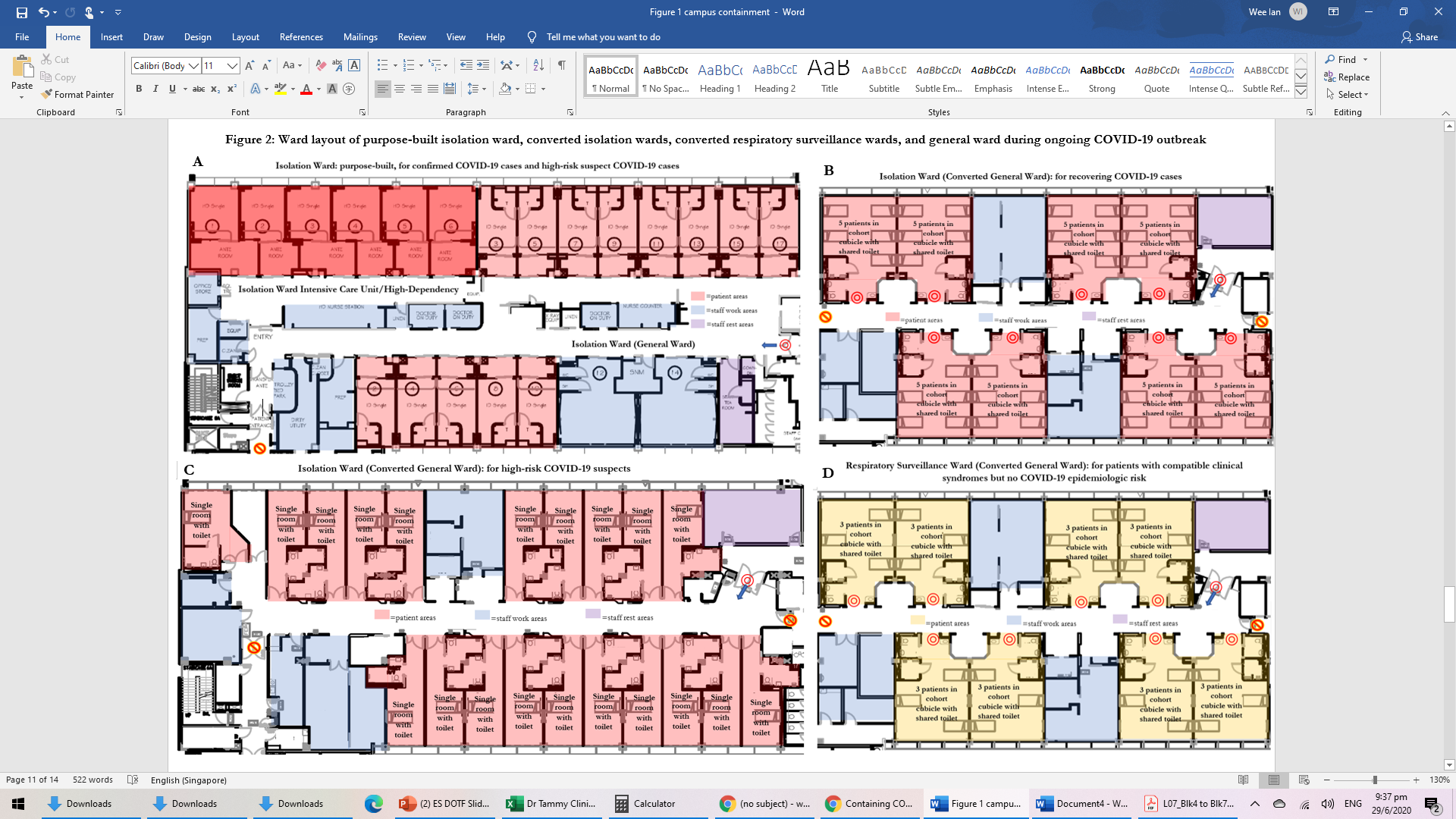


**NHC: National Heart Centre**

**SGH: Singapore General Hospital**

**Supplementary Figure 1: Details of solitary case of healthcare-associated adenoviral infection during COVID-19 pandemic; and number of death cases with PCR-proven adenoviral infections at a tertiary hospital in Singapore**

**Number of death cases with PCR-proven adenoviral infection, 2015-2020**

**B**

**Antechamber**

**Patient room**

**En-suite toilet**

**Purpose-built isolation ward**

Negative-pressure airborne isolation rooms, single occupancy, with en-suite toilet and antechamber; staff donned personal-protective-equipment prior to entry into antechamber

**Healthcare-associated adenoviral infection during COVID-19 pandemic**

Middle-aged returning traveller with concurrent COVID-19

- Duration of stay: 21 days
- Isolated in negative-pressure airborne-infection-isolation-room (AIIR) throughout admission
- No visitors allowed

Respiratory specimens on admission:

+ve for SARS-CoV-2, -ve for other viruses

Respiratory specimens on discharge:

-ve for SARS-CoV-2, +ve for adenovirus

Personal-protective-equipment in isolation ward:

Disposable gloves, gowns, eye protection, N95 respirators

(changed with every patient contact; no reuse)

**A**
