# Supplementary material for: Impact of infection prevention precautions on adenoviral infections during the coronavirus disease 2019 (COVID-19) pandemic: Experience of a tertiary-care hospital in Singapore
Source: Infect Control Hosp Epidemiol. 2020 Dec 10:1–2. doi: 10.1017/ice.2020.1365 (PMC8770837; doi:10.1017/ice.2020.1365)
Supplement: Supplementary file 1 [file icesup.zip › S0899823X20013653sup002.docx]

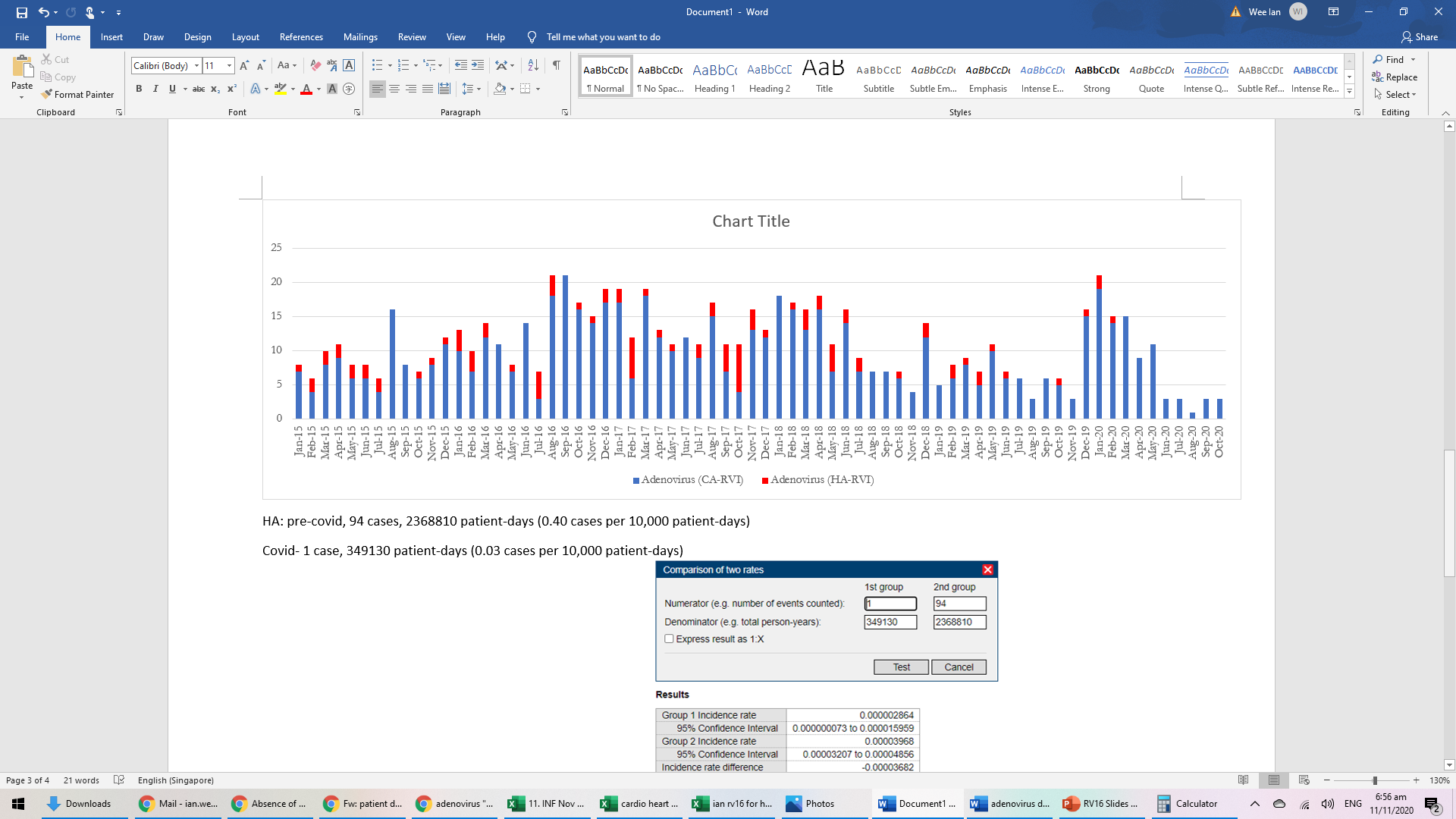


**Supplementary Figure 2: Trends in community-acquired (CA) and healthcare-associated (HA) adenoviral infections amongst all admissions to a tertiary hospital in Singapore**

**Trend in community-acquired (CA) and healthcare-associated (HA) adenoviral infections, 2015-2020, amongst all admissions**

Number of cases

COVID-19 pandemic
